# Positive Margin Location and Prostate Biopsy Route: A Consecutive Cohort Comparison of Transperineal and Transrectal Techniques

**DOI:** 10.3390/cancers18050849

**Published:** 2026-03-06

**Authors:** Abdullah Al-Khanaty, Kieran Sandhu, Marian S. Wettstein, Tess Howard, Modher Al-Shawi, William Nolan, Marlon Perera, Nathan Lawrentschuk, Cédric Poyet, Damien Bolton, Gregory Jack

**Affiliations:** 1 Division of Cancer Surgery, Peter MacCallum Cancer Centre, 305 Grattan Street, Melbourne, VIC 3000, Australia; 2 Department of Surgery, Austin Health, University of Melbourne, Melbourne, VIC 3084, Australia; 3 Department of Urology, Austin Health & Olivia Newton John Cancer Centre, Heidelberg, VIC 3084, Australia; 4 St Vincent’s Hospital, Melbourne, VIC 3065, Australia; 5 Department of Surgery, Royal Melbourne Hospital, University of Melbourne, Melbourne, VIC 3052, Australia; 6 Department of Urology, University Hospital of Zurich, 8091 Zurich, Switzerland

**Keywords:** prostate cancer, radical prostatectomy, positive surgical margins, transperineal prostate biopsy, transrectal prostate biopsy

## Abstract

**Background**: Transperineal (TP) prostate biopsy is increasingly used due to its superior infectious safety and enhanced sampling of anterior and apical prostate regions. Concerns have been raised that TP biopsy may induce greater post-biopsy fibrosis, particularly at the apex, potentially increasing positive surgical margin (PSM) rates at radical prostatectomy (RP). We evaluated whether biopsy technique influences overall or location-specific PSMs at RP. **Methods**: We performed a retrospective comparative cohort study of 1027 consecutive men who underwent TR biopsy and TP biopsy at our institution as we transitioned from TR to TP diagnostic technique. Patients from this cohort who were subsequently diagnosed with clinically localised prostate cancer and underwent RP were analysed. Clinical, biopsy, and pathological data were collected; all Gleason scores were standardised to Prognostic Grade Groups. PSMs were defined as tumour at the inked margin and categorised as apical, lateroposterior, or basal. Associations between biopsy technique and PSM outcomes were analysed using uni- and multivariable logistic regression. **Results**: Among 1027 biopsies, 260 men proceeded to RP, including 114 following TP biopsy and 146 following TR biopsy. Baseline pathological characteristics were similar between groups. Overall PSM rates did not differ between TP and TR cohorts (38.6 percent vs. 41.8 percent, *p* = 0.604). Margin location was also comparable at the apex (11.9 percent vs. 13.5 percent), lateroposterior (5.4 percent vs. 10.8 percent), and base (4.2 percent vs. 5.8 percent) respectively. In adjusted analyses controlling for age, PSA, grade, tumour stage, nodal status, and surgical approach, biopsy technique was not associated with overall PSMs (OR 0.70; 95 percent CI 0.39–1.24; *p* = 0.22) or with any location-specific PSMs. **Conclusions**: TP biopsy did not increase the risk of positive surgical margins at radical prostatectomy, either overall or at the apex. These findings do not support concerns that TP biopsy disrupts apical tissue planes or compromises oncologic outcomes. The results reinforce the safety of TP biopsy as a contemporary diagnostic standard, while highlighting the need for larger prospective studies to confirm these observations.

## Simple Summary

Transperineal (TP) prostate biopsy is increasingly used because it lowers infection risk and improves sampling of certain regions of the prostate compared with the traditional transrectal (TR) approach. However, concerns have been raised that TP biopsy might cause more scarring near the apex of the prostate, potentially increasing the chance of leaving microscopic cancer at the edge of the specimen during subsequent prostate removal surgery (radical prostatectomy), known as a positive surgical margin. In our study of over 1000 men during the transition from TR to TP biopsy, 260 went on to have surgery. We found no difference between the two biopsy methods in overall positive surgical margin rates or in specific margin locations. After adjusting for tumour features and surgical factors, biopsy technique was not associated with margin risk, supporting the oncologic safety of the transperineal approach.

## 1. Introduction

Prostate cancer is the most diagnosed cancer in men worldwide and a major source of morbidity, with more than 26,000 new diagnoses in Australia in 2024 and global incidence projected to almost double by 2040 [1,2,3].

Transrectal (TR) ultrasound-guided biopsy has been the foundation of prostate cancer diagnosis for several decades. Recently, the transperineal (TP) approach has undergone rapid international adoption, driven by its lower risk of post-biopsy sepsis, superior access to anterior and apical regions [4], and seamless integration with contemporary MRI-targeted and fusion-guided diagnostic pathways [5,6,7,8,9,10,11]. As TP biopsy becomes the dominant diagnostic pathway in many health systems, important questions have emerged regarding whether the route of biopsy may influence downstream surgical and oncologic outcomes.

A key consideration is that TP biopsy typically permits more extensive and systematic gland sampling than the TR approach and commonly involves a greater number of cores [12]. Studies have reported improved concordance between TP biopsy findings and final pathology at radical prostatectomy (RP), particularly for anterior or higher-grade tumours [13]. However, broader sampling and the concentrated needle trajectory through the apical capsule have raised theoretical concerns regarding apical haematoma biopsy-induced fibrosis around the urethral and apical edges of the prostate. The apical region represents a technically challenging aspect of RP, requiring meticulous identification of the urethro-prostatic interface, careful nerve preservation, and precise handling of continence structures. Even minor degrees of fibrosis, haematoma, or inflammatory change within these planes have the potential to obscure dissection landmarks, increase capsular tension, or predispose to focal capsular incision.

Based on these anatomic considerations, it has been hypothesised that TP biopsy may lead to increased post-biopsy fibrosis—particularly at the apex—and thereby increase the risk of positive surgical margins (PSMs) in this location, or shift their distribution toward apical locations. Some surgeons have expressed concern that disruption of the periapical tissue planes following TP biopsy may reduce the clarity of capsular margins encountered during RP, complicating apical dissection, compromising nerve preservation, or increasing the likelihood of focal capsular breach. Given that PSMs, especially at the apex, are associated with higher biochemical recurrence rates and an increased likelihood of requiring adjuvant radiotherapy, determining whether biopsy technique influences this outcome carries meaningful clinical implications.

Despite the biological plausibility of this hypothesis, evidence remains limited and inconsistent. Several published series report no association between biopsy route and PSM rates, while others describe subtle histological fibrosis after TP biopsy without a demonstrable surgical impact. Importantly, very few studies have examined PSM location, even though the proposed mechanism of TP-related fibrosis is anatomically focal rather than global. High-quality comparative data evaluating margin distribution after TP versus TR biopsy are scarce, and definitive conclusions cannot be drawn from the existing literature.

Our institution provides an ideal setting to address this question. In December 2015, our diagnostic pathway transitioned from exclusively TR biopsy to exclusively TP biopsy, creating two clearly defined, internally consistent matched cohorts. This enables a direct comparison of surgical and pathological outcomes within a stable institutional environment, surgeon cohort, and pathology reporting framework.

The objective of this study was to determine whether TP biopsy, compared with TR biopsy, is associated with higher overall PSM rates or with shifts in anatomical PSM distribution at the time of radical prostatectomy. We hypothesised that TP biopsy may be associated with increased apical PSMs due to a higher density of needle tracts and potential localised fibrosis in these regions.

## 2. Materials and Methods

### 2.1. Study Design and Patient Identification

This study was designed as a retrospective comparative cohort analysis conducted at a single Australian tertiary academic centre. All men who underwent a prostate biopsy between September 2013 and July 2017 were identified using institutional electronic records. During this timeframe, the diagnostic pathway for prostate cancer at our institution underwent a transition from TR biopsy to TP biopsy. This created two identical matched cohorts that allowed a natural comparison between the biopsy techniques without selective allocation or variation in other surgical parameters such as surgeon or technique.

Men were eligible for inclusion if they had histologically confirmed prostate cancer on biopsy, clinically localised disease, and proceeded to radical prostatectomy at the same institution. Patients managed with primary radiotherapy, active surveillance, focal therapies, or those who underwent prostatectomy elsewhere were excluded. Only men with complete clinical and pathological data were included in the final analysis.

### 2.2. Biopsy and Surgical Techniques

The TR approach was performed under anaesthesia with transrectal ultrasound guidance, sampling the prostate through the rectal plane, taking an 8–24 core sextant template, using a Bard Max-Core™ Disposable Core Biopsy Instrument(Macquarie Park, NSW, Australia ) 18 G × 20 cm. After the institutional shift to TP biopsy, the procedures were performed under general anaesthesia and identical transrectal ultrasound guidance, taking an 18–24 core sextant template through the perineum, using the same Bard Max-Core™ Disposable Core Biopsy 18 G × 20 cm Instrument.

All radical prostatectomies were performed by supervised trainee urologic surgeons using either a retropubic open technique or an extraperitoneal laparoscopic approach, depending on surgeon preference. Specimens were processed and reported by dedicated genitourinary pathologists following consistent institutional protocols. Surgical margin status was determined using standard definitions, and tumour grade was standardised to the contemporary Prognostic Grade Group (PGG) system.

### 2.3. Data Collection and Statistical Analysis

Clinical, demographic, and biopsy-related details were obtained from medical records and original pathology reports, while final pathological findings from the radical prostatectomy specimen were extracted from definitive histological assessments. All original Gleason scores were converted to the PGG classification according to established criteria [14,15]. A positive surgical margin (PSM) was defined as the presence of tumour cells at the inked surface of the specimen, and each PSM was assigned an anatomical location categorised as apical/anterior, lateroposterior, or basal. Margin location categories were not mutually exclusive; a given patient could have positive margins at more than one anatomical site.

Statistical analyses were performed using SPSS version 24.0 and R version 3.4.4. Baseline differences between the TP and TR cohorts were evaluated using chi-square testing for categorical variables and the Mann–Whitney U test for continuous nonparametric variables such as age, PSA, and number of biopsy cores. Associations between biopsy technique and overall or site-specific PSMs were examined using univariable logistic regression, followed by multivariable logistic regression adjusting for age, PSA, tumour grade, pathological stage, nodal status, and surgical approach. Adjustments were limited in models with smaller numbers of events to avoid overspecification. All *p*-values were two sided, with statistical significance set at <0.05.

## 3. Results

### 3.1. Cohort Identification and Baseline Characteristics

Between September 2013 and July 2017, 1027 consecutive prostate biopsies were performed at our institution. Of these, 546 biopsies (53.2 percent) were performed using the transrectal (TR) approach and 481 biopsies (46.8 percent) were performed using the transperineal (TP) approach. A total of 260 men with biopsy-proven, clinically localised prostate cancer proceeded to radical prostatectomy at the same institution and were included in the final analysis. Among these, 114 men (43.7 percent) had undergone TP biopsy and 146 (56.3 percent) had undergone TR biopsy.

Baseline clinical and pathological characteristics of the cohort are summarised in Table 1. Men in the TP cohort were slightly older and had marginally higher PSA levels at diagnosis. As expected, more biopsy cores were obtained in the TP group than in the TR group (median 24 vs. 14 cores, *p* < 0.001). Despite these differences, the groups were similar in final pathological grade distribution, tumour stage, and nodal status following radical prostatectomy. Surgical approach differed between the cohorts, with open prostatectomy used more frequently in the TP group and laparoscopic prostatectomy more common in the TR group (70.2 percent vs. 53.4 percent, *p* = 0.006).

### 3.2. Positive Surgical Margin Rates and Distribution

**Figure 1:**
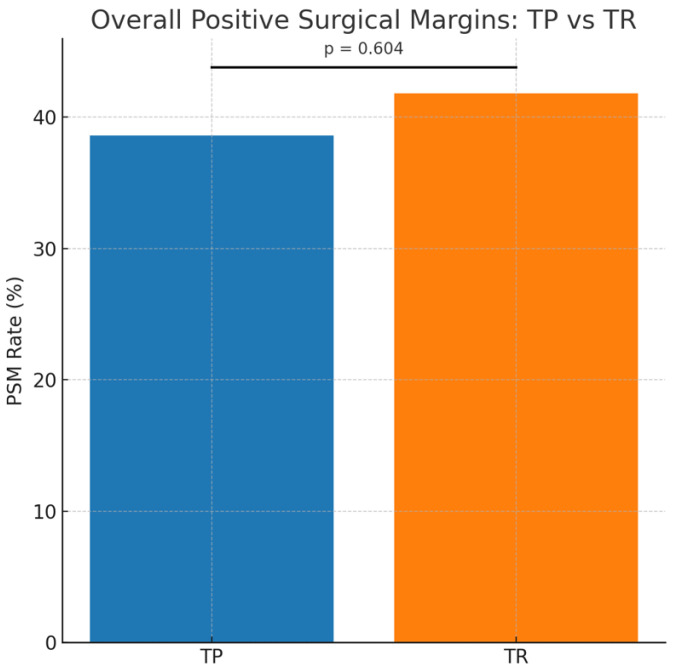
Overall positive surgical margin (PSM) rates following radical prostatectomy according to biopsy technique. Bar chart comparing overall PSM rates between men diagnosed with transperineal biopsy (TP; *n* = 114) and transrectal biopsy (TR; *n* = 146). Overall PSM rates were 38.6 percent for TP and 41.8 percent for TR, with no significant difference between groups (*p* = 0.604).

Overall, 105 of the 260 men (40.4 percent) had a positive surgical margin (PSM) at radical prostatectomy. The proportion of patients with a PSM did not differ significantly between biopsy groups: 38.6 percent in the TP cohort and 41.8 percent in the TR cohort (*p* = 0.604) (Figure 1).

**Figure 2:**
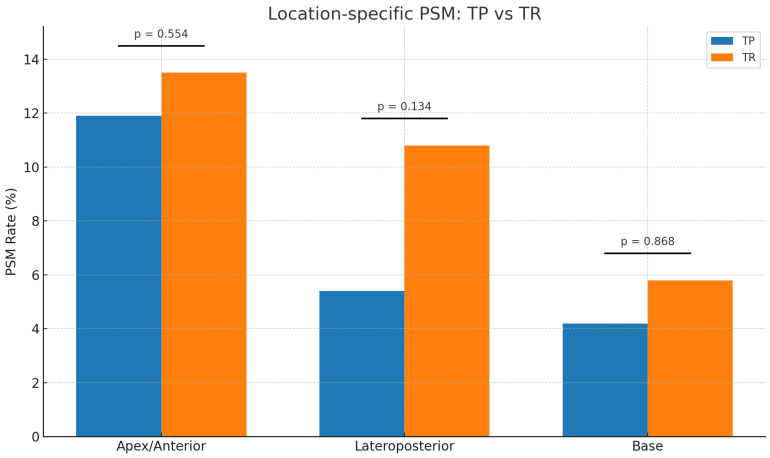
Location-specific positive surgical margin (PSM) rates stratified by biopsy technique. Apex/anterior, lateroposterior, and basal PSM rates are shown for men diagnosed using transperineal biopsy (TP; *n* = 114) and transrectal biopsy (TR; *n* = 146). No significant differences were observed between TP and TR biopsy for apex/anterior (11.9 percent vs. 13.5 percent; *p* = 0.554), lateroposterior (5.4 percent vs. 10.8 percent; *p* = 0.134), or basal (4.2 percent vs. 5.8 percent; *p* = 0.868) margins.

Margin location was also evaluated according to biopsy technique and is reported in Table 2. PSMs at the apex region were observed in 25.4 percent of the entire cohort, with comparable rates between TP and TR biopsy groups (11.9 percent vs. 13.5 percent, *p* = 0.554). Lateroposterior PSMs occurred in 16.2 percent of patients overall, and although these were numerically lower in the TP cohort (5.4 percent vs. 10.8 percent), the difference did not reach statistical significance (*p* = 0.134). PSMs at the base were infrequent and similar between groups (4.2 percent vs. 5.8 percent, *p* = 0.868) (Figure 2). Because margin location categories were not mutually exclusive, the sum of location-specific margins exceeds the overall PSM count.

The distribution of PSMs across anatomical regions is illustrated in Figure 1, demonstrating similar patterns in margin location when comparing the TP and TR cohorts.

### 3.3. Regression Analyses

Univariable and multivariable logistic regression analyses were performed to assess whether biopsy technique was associated with PSM occurrence, both overall and by specific anatomical location.

In unadjusted analysis, TP biopsy was not associated with overall PSM risk (odds ratio [OR] 0.88; 95 percent confidence interval [CI] 0.53–1.44; *p* = 0.60). After adjustment for age, PSA, tumour grade, pathological stage, nodal status, and surgical approach, the association remained nonsignificant (adjusted OR 0.70; 95 percent CI 0.39–1.24; *p* = 0.22).

Similarly, biopsy technique was not associated with PSMs at the apex, lateroposterior, or base margins in either univariable or multivariable models. For apical PSMs, the adjusted OR was 1.11 (95 percent CI 0.60–2.07; *p* = 0.74). For lateroposterior margins, the adjusted OR was 0.62 (95 percent CI 0.29–1.28; *p* = 0.20), and for base margins the adjusted OR was 0.98 (95 percent CI 0.40–2.30; *p* = 0.96). Full regression outputs are presented in Table 3.

## 4. Discussion

In this consecutive comparative cohort study, we evaluated whether biopsy technique—TP versus TR—influences overall or site-specific PSM rates at RP. The role of TP prostate biopsy has evolved substantially in recent years and has, in many centres, supplanted the TR approach for two principal reasons. First, TP biopsy offers improved cancer detection through more efficient and systematic sampling, enabling more accurate identification of prostate cancer, particularly within the anterior regions of the prostate [9,16]. Second, TP biopsy is associated with a near-zero risk of post-biopsy sepsis, in contrast to TR biopsy, for which rising infection rates have been reported, largely driven by an increasing prevalence of multidrug-resistant rectal flora [17,18,19].

Despite these advantages, TP biopsy has recognised limitations. The procedure is typically more time-consuming and has traditionally required general anaesthesia in an operating theatre setting [5,20,21], although this is evolving with the increasing adoption of local anaesthetic techniques [22,23]. In addition, higher rates of acute urinary retention have been reported compared with TR biopsy [24]. Nevertheless, TP biopsy allows more extensive sampling strategies, including template, saturation, and targeted approaches, without compromising its favourable infectious safety profile [25].

Although TP biopsy traverses apical tissue planes, giving rise to theoretical concerns about needle-tract fibrosis and altered dissection planes in the apex during radical prostatectomy, our data did not demonstrate any significant difference in either overall PSMs or the anatomical distribution of margins. These findings remained consistent after multivariable adjustment for clinical and pathological variables, suggesting that biopsy route does not exert a clinically meaningful influence on oncologic quality at RP.

The biological rationale for this hypothesis stems from earlier descriptions of post-biopsy tissue changes. Barzell and Melamed [26] reported substantial periprostatic fibrosis following intensive 3D template mapping biopsies, occasionally limiting neurovascular bundle preservation, although these procedures involved far greater sampling density than contemporary diagnostic TP biopsy and are not reflective of modern practice. In contrast, Huo et al. [27] demonstrated that nerve-sparing surgery could be successfully performed following transperineal template biopsy despite comparable degrees of periprostatic fibrosis at radical prostatectomy, and Dimmen et al. [28] similarly reported no technical difficulties during robotic-assisted radical prostatectomy after TP biopsy.

Only a limited number of studies have directly compared radical prostatectomy outcomes following different biopsy routes. Yao et al. [29] reported no difference in positive surgical margin (PSM) rates between TR and TP biopsy cohorts (21.2% vs. 21.9%, *p* = 0.895), with consistent findings on subgroup analysis of pT2 and pT3 disease. Although increased periprostatic fibrosis was observed histologically following TP biopsy, this did not translate into higher PSM rates or adverse early oncologic outcomes. Similarly, Wadhwa et al. [30] evaluated 181 men undergoing robotic prostatectomy and demonstrated no significant differences in margin rates or biochemical recurrence between TR and TP biopsy groups, despite hypothesising potential apical fibrosis with TP biopsy. Hossack et al. [16] analysed 1132 men (414 TP, 718 TR) and found comparable tumour characteristics overall; while PSM rates were higher in TP patients with pT2 disease, this was attributed to an institutional robotic learning curve rather than biopsy technique. Notably, earlier studies did not consistently incorporate multivariable adjustment, limiting causal inference. Collectively, contemporary data suggest that any fibrosis induced by modern TP biopsy is insufficient to compromise oncologic outcomes following radical prostatectomy.

Although no associations in our study reached statistical significance, the directionality of odds ratios warrants attention. Apical/anterior margins demonstrated an odds ratio greater than 1—aligned with the theoretical trajectory of TP needle tracts—while lateroposterior and basal margins trended below unity. The wide confidence intervals associated with these estimates indicate that the study may have been underpowered to detect small but potentially meaningful differences in location-specific margins. Larger, multi-centre datasets may therefore be required to determine whether these subtle trends reflect true biological effects or random variation. Nevertheless, the absence of a consistent or strong signal, combined with alignment with the broader literature, supports the conclusion that TP biopsy does not adversely affect surgical margin outcomes.

The overall positive surgical margin rate in our cohort is higher than that reported in some contemporary high-volume robotic series. However, this must be interpreted in the context of case mix and practice setting. Approximately half of patients in our study had ≥pT3 disease, a well-established independent predictor of margin positivity, with reported rates frequently exceeding 40–50% in extraprostatic extension. In addition, 60% of procedures were performed via an open approach, and all cases were conducted within a public hospital training environment with registrar involvement. Our institution also serves as a high-risk referral centre, further enriching the cohort for adverse pathological features. Collectively, these factors reflect a real-world surgical population rather than a highly selected private or single-surgeon robotic series. As such, the observed margin rates are consistent with the underlying disease severity and operative context, and importantly, did not differ between biopsy techniques.

The difference in surgical approach between groups reflects a transitional institutional period. The earlier TR era included a greater proportion of laparoscopic procedures, whereas during the later TP era, open prostatectomy was more frequently utilised within a public hospital training environment. Surgical approach was recorded according to the initial operative modality commenced, and conversion rates were not separately documented.

This study has several important strengths. The institutional transition from TR to TP biopsy created two discrete diagnostic eras, reducing the selection bias often associated with clinician-driven biopsy choice, as biopsy route was determined by institutional practice rather than patient characteristics. Conducting the study within a single centre ensured relative consistency in surgical technique, specimen handling, and pathological reporting, thereby minimising inter-surgeon and inter-observer variability. The comparatively large number of men proceeding to radical prostatectomy strengthens internal validity. Importantly, our anatomically granular analysis of positive surgical margins—evaluating apical, lateroposterior, and basal locations separately rather than overall PSM rates alone—directly addresses the primary biological concern surrounding TP biopsy, namely the potential for apical fibrosis and disruption of vulnerable dissection planes. Furthermore, the use of multivariable logistic regression allowed adjustment for established confounders, representing an analytic strength not consistently present in earlier comparative studies.

This study has limitations. Its retrospective design introduces potential confounding despite multivariable adjustment. As a single-centre cohort, results may not be generalisable to all practice settings. Differences in surgical approach (open versus laparoscopic) may have influenced outcomes, although this was accounted for in regression models. The interval between biopsy and radical prostatectomy was not consistently available in the retrospective dataset and therefore could not be analysed; given that the central hypothesis relates to potential post-biopsy fibrosis, variation in time to surgery may theoretically influence tissue healing and dissection planes. Long-term oncologic outcomes such as biochemical recurrence and metastasis-free survival were also not assessed due to heterogeneous follow-up duration and may yield further insight into the clinical significance of subtle differences in margin status. Margin location categories were not mutually exclusive, and some patients had positive margins at more than one anatomical site; however, due to limitations of the retrospective data extract, we were unable to quantify the proportion of patients with multiple positive margin locations. In addition, total biopsy core number was not consistently available across the cohort and therefore could not be incorporated into adjusted models. Given that biopsy core number was closely linked to biopsy route during the institutional transition period, this represents a potential source of residual confounding. Finally, despite a reasonable sample size, subgroup analyses—particularly for specific anatomical margins—may remain underpowered, as reflected by wide confidence intervals.

Future studies should align with the evolution of contemporary diagnostic pathways, particularly MRI-fusion and peri-lesional transperineal biopsy techniques. These approaches differ from traditional systematic biopsy in sampling density, trajectory, and focality, and their downstream impact on periprostatic tissue integrity remains incompletely characterised. Prospective investigations incorporating MRI both before and after biopsy could clarify whether targeted transperineal sampling produces focal haemorrhage or fibrosis that might influence subsequent surgical dissection planes. With the widespread adoption of robotic prostatectomy in Australia, future cohorts will also be better positioned to control for surgical platform and operative technique. Large, multi-centre datasets incorporating granular anatomical margin mapping, integrated surgical video analysis, and longer-term oncologic follow-up will be essential to definitively determine whether subtle biopsy-related tissue effects exist.

## 5. Conclusions

In this consecutive comparative cohort study, transperineal prostate biopsy was not associated with higher apical positive surgical margin rates at radical prostatectomy when compared with the transrectal approach. Despite theoretical concerns regarding biopsy-related apical fibrosis, our findings do not demonstrate any adverse impact of the transperineal technique on surgical margin outcomes. These results support the oncologic safety of transperineal biopsy within contemporary diagnostic pathways. Larger, prospective, and multi-centre studies are warranted to confirm these observations and further clarify whether subtle differences in margin patterns exist.

## Figures and Tables

**Table 1:** Characteristics of patients undergoing radical prostatectomy (*n* = 260).

Variable		Overall	TP	TR	*p*-Value *
Number of patients		260 (100)	114 (43.7)	146 (56.3)	
Age at diagnosis (years)		64 (45–74)	65 (45–74)	63 (47–73)	**0.022**
PSA (ug/L)		7.17 (1.90–31.90)	7.88 (3.30–31.90)	7.00 (1.90–25.00)	**0.025**
Number of biopsy cores taken		18 (8–50)	24 (13–50)	14 (8–32)	**<0.001**
*Final Pathology*					
Prognostic Grade Group (Gleason Score)					0.179
	1	20 (7.7)	4 (3.5)	16 (11)	
	2	124 (47.7)	61 (53.5)	63 (43.2)	
	3	66 (25.4)	26 (22.8)	40 (27.4)	
	4 or 5	50 (19.2)	23 (20.2)	27 (18.5)	
Tumour stage					0.240
	≤pT2	128 (49.2)	62 (54.4)	66 (45.2)	
	≥pT3	130 (49.7)	51 (44.7)	79 (54.1)	
Nodal stage					0.760
	cN0	163 (62.7)	71 (62.3)	92 (63)	
	pN0	84 (32.3)	36 (31.6)	48 (32.9)	
	pN1	11 (4.2)	6 (5.3)	5 (3.4)	
Surgical margins					0.604
	NSM	155 (59.6)	70 (61.4)	85 (58.2)	
	PSM	105 (40.4)	44 (38.6)	61 (41.8)	
Radical prostatectomy					**0.006**
	open	158 (60.8)	80 (70.2)	78 (53.4)	
	laparoscopic	102 (39.2)	34 (29.8)	68 (46.6)	

* Chi-square, for nonparametric values (age, PSA, prostate volume) Mann–Whitney U test. Data are presented as median (range) or number (percent). Abbreviations: PSA = prostate-specific antigen, TP = transperineal, TR = transrectal.

**Table 2:** Occurrence and location of PSM (*n* = 260).

Variable	Overall (N = 260)	TP (N = 114)	TR (N = 146)	*p*-Value *
PSM	105 (40.4)	44 (38.6)	61 (41.8)	0.604
Apex	66 (25.4)	31 (11.9)	35 (13.5)	0.554
Lateroposterior	42 (16.2)	14 (5.4)	28 (10.8)	0.134
Base	26 (10)	11 (4.2)	15 (5.8)	0.868

* Chi-square. Data are presented as number (percent). Abbreviations: PSM = positive surgical margin, TP = transperineal, TR = transrectal.

**Table 3:** Univariable and multivariable logistic regression analysis investigating the association of biopsy technique and PSM overall and at different locations.

Variable	OR (95% CI)	*p*-Value
	Outcome PSM (Overall)	
TP vs. TR biopsy (unadjusted)	0.88 (0.53–1.44)	0.60
TP vs. TR biopsy (adjusted ^1^)	0.7 (0.39–1.24)	0.22
	Outcome Apex PSM	
TP vs. TR biopsy (unadjusted)	1.33 (0.75–2.35)	0.33
TP vs. TR biopsy (adjusted ^2^)	1.11 (0.60–2.07)	0.74
	Outcome Lateroposterior PSM	
TP vs. TR biopsy (unadjusted)	0.59 (0.29–1.16)	0.14
TP vs. TR biopsy (adjusted ^3^)	0.62 (0.29–1.28)	0.20
	Outcome Base PSM	
TP vs. TR biopsy (unadjusted)	0.93 (0.40–2.11)	0.87
TP vs. TR biopsy (adjusted ^3^)	0.98 (0.40–2.30)	0.96

Abbreviations: PSM = positive surgical margin, TP = transperineal, TR = transrectal. ^1^ Adjusted for age, PSA, Gleason score (Gleason 6, Gleason 7 and ≥Gleason 8), tumour stage (≤pT2 vs. ≥pT3), nodal stage (c/pN0 vs. pN1) and surgery (open vs. laproscopic). ^2^ Adjusted for age, PSA, Gleason score (Gleason ≤ 7 vs. ≥Gleason 8), tumour stage (≤pT2 vs. ≥pT3) and surgery (open vs. laproscopic). ^3^ Adjusted for Gleason score (Gleason ≤ 7 vs. ≥Gleason 8), tumour stage (≤pT2 vs. ≥pT3) and surgery (open vs. laproscopic).

## Data Availability

Data will be provided upon request.
